# *EPHA4* haploinsufficiency is responsible for the short stature of a patient with 2q35-q36.2 deletion and Waardenburg syndrome

**DOI:** 10.1186/s12881-015-0165-2

**Published:** 2015-04-11

**Authors:** Chuan Li, Rongyu Chen, Xin Fan, Jingsi Luo, Jiale Qian, Jin Wang, Bobo Xie, Yiping Shen, Shaoke Chen

**Affiliations:** Genetic and Metabolic Central Laboratory, Guangxi Maternal and Child Health Hospital, Nanning, GuangXi China; Department of Laboratory Medicine, Department of Pathology, Boston Children’s Hospital, Harvard Medical School, Boston, MA 02115 USA; Claritas Genomics, Boston, MA 02139 USA

**Keywords:** Chromosomal microarray, 2q35-q36.2, *PAX3*, Waardenburg syndrome, *EPHA4*, Short stature

## Abstract

**Background:**

Waardenburg syndrome type I (WS1), an auditory-pigmentary genetic disorder, is caused by heterozygous loss-of-function mutations in *PAX3*. Abnormal physical signs such as dystopia canthorum, patchy hypopigmentation and sensorineural hearing loss are common, but short stature is not associated with WS1.

**Case presentation:**

We reported a 4-year and 6 month-old boy with a rare combination of WS1 and severe short stature (83.5 cm (−5.8SD)). His facial features include dystopia canthorum, mild synophrys, slightly up-slanted palpebral fissure, posteriorly rotated ears, alae nasi hypoplasia and micrognathia. No heterochromia was noticed. He had a normal intelligence quotient and hearing. Insulin-like growth factor-1 (IGF-1) was 52.7 ng/ml, lower than the normal range (55 ~ 452 ng/ml) and the peak growth hormone level was 7.57 ng/ml at 90 minutes after taking moderate levodopa and pyridostigmine bromide. The patient exhibited a good response to human growth hormone (rhGH) replacement therapy, showing a 9.2 cm/year growth rate and an improvement of 1 standard deviation (SD) of height after one year treatment. CMA test of patient’s DNA revealed a 4.46 Mb *de novo* deletion at 2q35-q36.2 (hg19; chr2:221,234,146-225,697,363).

**Conclusions:**

*PAX3* haploinsufficiency is known to cause Waardenburg syndrome. Examining overlapping deletions in patients led to the conclusion that *EPHA4* is a novel short stature gene. The finding is supported by the *splotch-retarded* and *epha4* knockout mouse models which both showed growth retardation. We believe this rare condition is caused by the haploinsufficiency of both *PAX3* and *EPH4* genes. We further reported a growth response to recombinant human growth hormone treatment in this patient.

## Background

Waardenburg syndrome (WS) is a group of auditory-pigmentary genetic disorders caused by neural crest development defects. Mutations in *PAX3*, a transcription factor belonging to the family of paired-box-containing proteins, causes WS type 1 (WS1, OMIM # 193500) and type 3 (WS3, OMIM # 148820). WS1 and WS3 are characterized by dystopia canthorum, a lateral displacement of inner canthus of the eyes. This feature is highly penetrant for WS1, can be objectively determined by measuring the W index^a^ [1]. Hearing loss, pigmentary disturbances of the iris, hair and skin are other common features of Waardenburg syndrome with variable penetrance. Clinically, Waardenburg syndrome can be diagnosed based on the presence of two major features or one major feature plus two minor features [2]. Major features are sensorineural hearing loss, iris pigmentary abnormality, hair hypopigmentation, dystopia canthorum (W index > 1.95) and affected first-degree relative. Minor features include skin hypopigmentation, synophrys, broad nasal root, hypoplastic alae nasi, and premature graying of the hair. Short stature is not associated with WS1. WS3, sometimes called Klein-Waardenburg syndrome, includes abnormalities of the upper limbs in addition to dystopia canthorum, hearing loss and changes in pigmentation. About 90% of pathogenic variants in *PAX3* are nucleotide level alterations detectable by sequencing, whereas an additional 10% of WS1 are caused by large deletions involving part or the whole *PAX3* gene [3]. These variants may be detected by karyotyping [4,5], FISH [6,7] and MLPA [8,9]. Recently chromosomal microarray (CMA) analysis has allowed for a more accurate delineation of the deletion interval and genes involved, allowing for better genotype-phenotype correlation study.

## Case presentation

**Figure 1:**
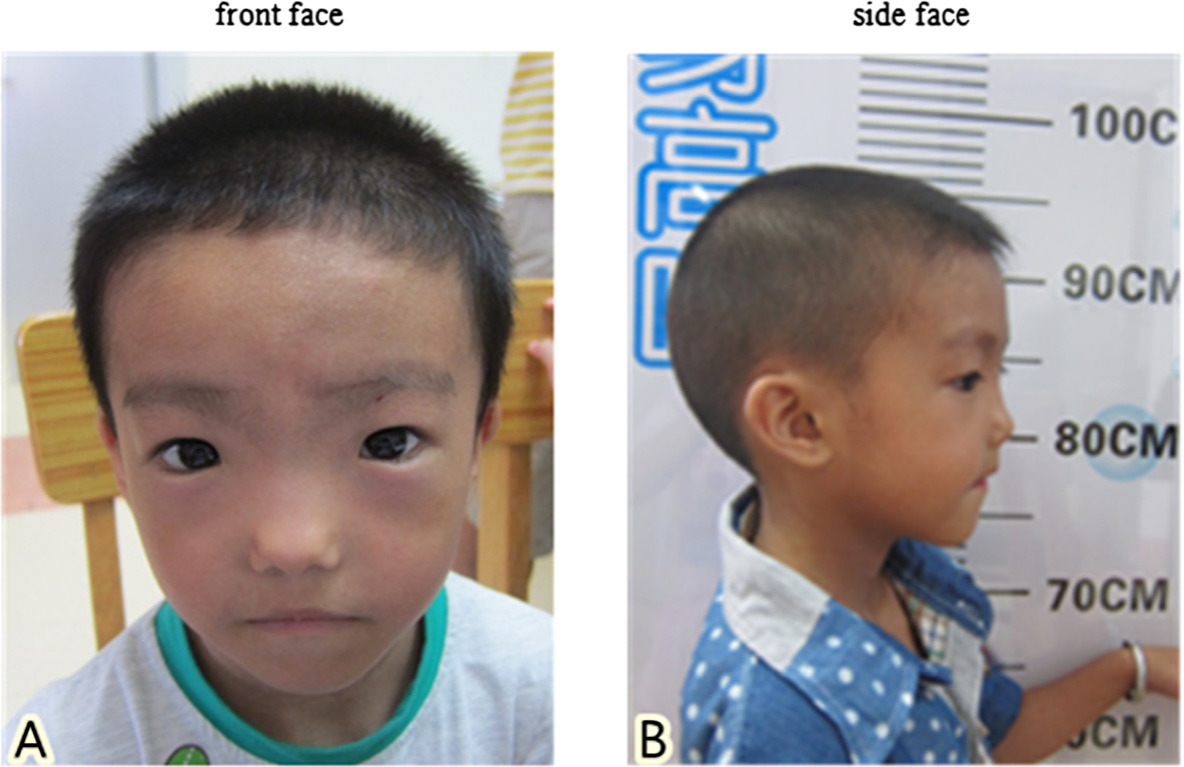
Characteristic facial feature of the patient. **A** and **B** are the front and side views of the patient. Notable features include ocular hypertelorism, dystopia canthorum, short philtrum and wide based nose root with hypoplasia alae nasi.

Here we report a 4-year and 6 month-old Chinese boy with a rare combination of Waardenburg syndrome type 1 and severe short stature. The patient was the first child of a non-consanguineous marriage. He had severe short stature (83.5 cm (−5.8SD)) and poor weight gain (10 kg (−4.1 SD)). Both parents had short stature but are healthy: father’s height is 153 cm (−3.2 SD) and mother’s height is 148 cm (−2.3SD). Patient’s short stature is proportionate. His facial features included dystopia canthorum (W index = 2.31), mild synophrys, slightly up-slanted palpebral fissure, posteriorly rotated ear, alae nasi hypoplasia and micrognathia (Figure 1a and b). He has normal intelligence and hearing. No heterochromia or other pigmentation anomalies was noticed. Physical examination did not find any other abnormalities. The patient met the clinical diagnostic criteria of WS1. Routine blood, urine tests as well as liver and renal function tests were all normal. Endocrine tests for TSH, FT3, FT4, FSH, LH and insulin were normal. Insulin-like growth factor-1 (IGF-1) was 52.7 ng/ml, lower than the normal range (55 ~ 452 ng/ml). The peak growth hormone level was 7.57 ng/ml at 90 minutes after taking moderate levodopa and pyridostigmine bromide. Brain MRI revealed a pituitary gland about 3.2 cm long without abnormal morphology. Bone age based on left hand X-ray is 1.5 years.

**Figure 2:**
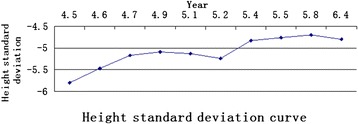
Height standard deviation curve during replacement therapy with rhGH.

The patient underwent recombinant human growth hormone (rhGH) replacement therapy for over a year. The daily dosage was 0.11 IU/kg. Growth velocity and side-effect profile were monitored for at regular intervals (Table 1). The patient exhibited a good response to rhGH treatment. He showed a 9.2 cm/year growth rate and an improvement of 1 SD of height after one year treatment (Figure 2).

**Table 1:** **Efficacy and side-effect of the rhGH treatment**

**Course of GH treatment**	**HT (cm)**	**HT SDS (SDS)**	**Body weight (kg)**	**Bone age (y)**	**Drug dose (IU/kg)**	**IGF-1 (ng/ml)**	**Side-effect**
prior treatment	83.5	−5.80	10.0	1.5	-	52.7	-
1 month of therapy	85.3	−5.47	10.0	-	0.11	75.8	N
3 month of therapy	87.0	−5.17	10.5	-	0.11	-	N
6 month of therapy	88.3	−5.09	11.0	2.0	0.10	99.1	N
7 month of therapy	89.5	−5.13	11.5	-	0.10	-	N
8 month of therapy	89.9	−5.24	10.5	-	0.11	-	N
11 month of therapy	92.2	−4.83	11.5	-	0.12	-	N
13 month of therapy	93.3	−4.76	11.0	2.0	0.12	128.0	N
15 months of therapy	94.8	−4.70	11.5	-	0.12	-	N
9 months out of therapy	98.1	−4.80	12.0	-	0.12	-	N

**Figure 3:**
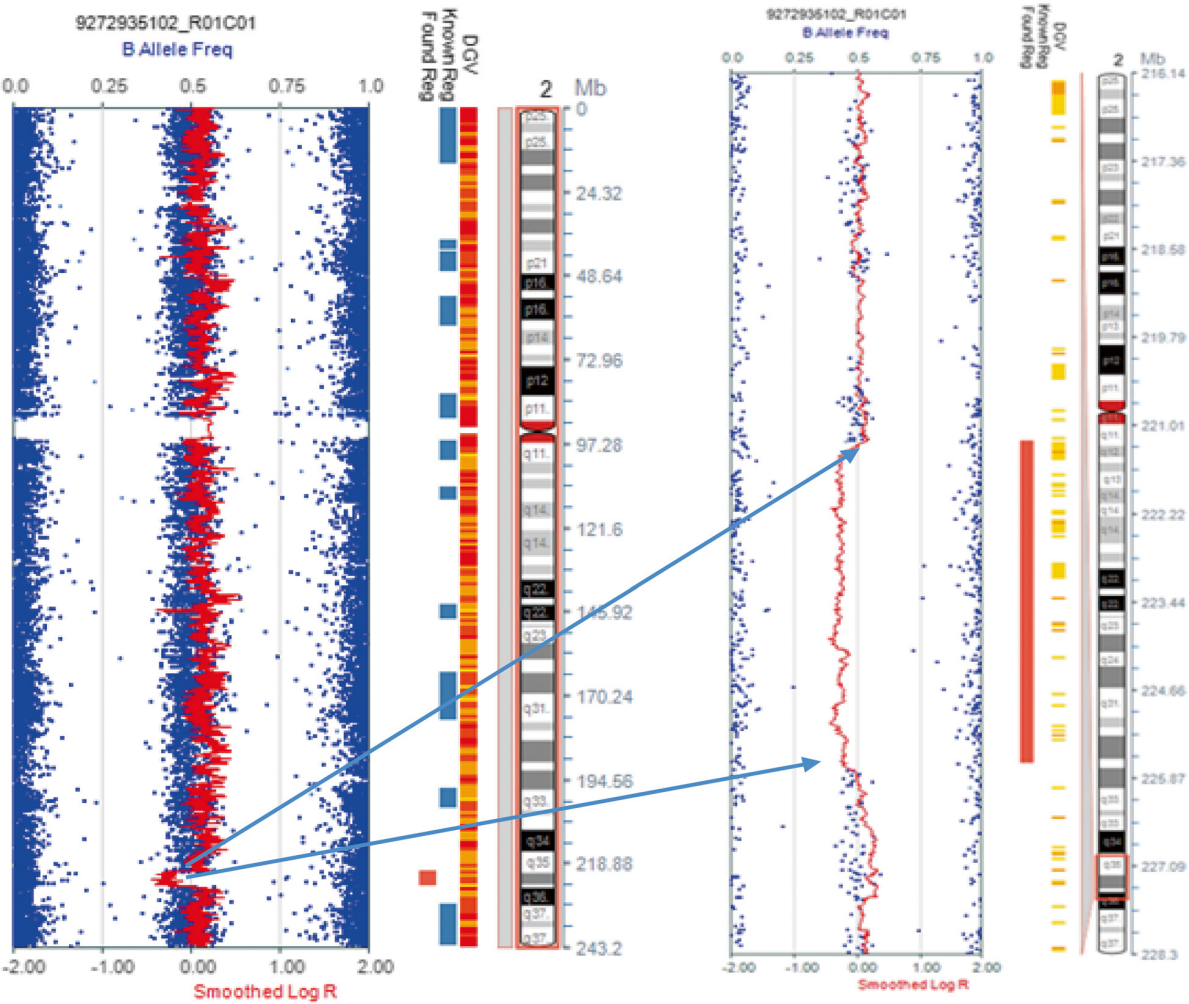
Array profile of the deleted region at 2q35q36.2. The left panel is the whole chromosome view; the right panel is a zoomed-in view of the deleted region demarcated by the two arrows and a vertical red bar.

CMA test of patient’s DNA using illumina Human SNP cyto-12 array revealed a 4.46 Mb *de novo* deletion at 2q35-q36.2 (chr2:221,234,146-225,697,363) (hg19, Figure 3). The deletion involved the whole *PAX3* gene which is responsible for the Waardenburg syndrome phenotype and neighboring genes including *EPHA4*.

**Figure 4:**
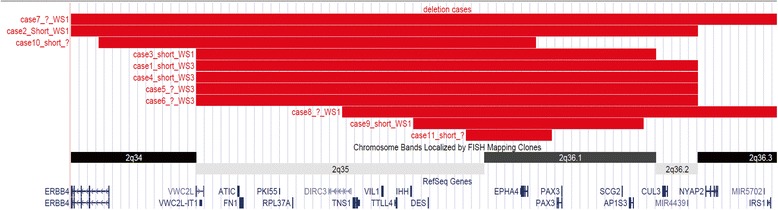
Previously published cases with overlapping deletion at 2q35-q36.2. (Each horizontal red bar represents the deletion interval of a case. The origins of these cases are listed in Table 2).

We evaluated previously published cases of overlapping deletions with our case’s (Table 2 and Figure 4). We noticed that two thirds of deletion cases reported short stature or growth retardation as one of the phenotypic features when *EPHA4* gene was involved in the deletions. The remaining cases did not provide height information. The animal model supported the notion that the *EPHA4* deletion is responsible for short stature. The Sp^r^ mutant was created by X-ray mutagenesis and characterized by a cytogenetically detectable deletion of band C4 on mouse chromosome 1. Heterozygous mice displayed white spotting of the belly, tail and feet, equivalent pigmentary features of WS. They also had persistent growth retardation throughout their development [10]. The deletion is approximately syntenic to human chr2:218,449,525-232,459,056 region, both *Pax3* and *Epha4* were involved in the deletion. The other *Splotch* mutants caused by missense mutation (*Sp*^*d*^) [11] or splicing mutation (*Sp*) [12] in *Pax3* gene do not exhibit growth retardation, suggesting genes adjacent to *Pax3* are responsible for the growth retardation phenotype. The most important evidence came from the recent *epha4* knockout mouse model. Both heterozygous and homozygous *epha4* knockout mice showed significant postnatal growth retardation in a dose dependent manner [13]. So far only one patient has been reported to carry a deletion involving only the *EPHA4* gene (DECIPHER 282314). It was a *de novo* deletion. The available phenotype of this patient involves the shortening of digital bones such as short 1st metacarpal, short distal phalanx of hallux, short distal phalanx of the 2nd finger, short distal phalanx of the thumb, short distal phalanx of toe and short first metatarsal. All these phenotypes were absent from either of the parents. The height information was not available for this patient. Due to their close proximity on chromosome 2, *EPHA4* and *PAX3* are often co-deleted as the cases shown in Figure 4. Most of them had reported short stature as a clinical feature [4-6,8,9,14-17]. In addition to *EPHA4* and *PAX3*, there are about 10 more OMIM genes involved at the deletion interval of our patient, three are associated with human diseases (AP1S3 is associated with the susceptibility to pustular psoriasis-15; MRPL44 is likely associated with Combined oxidative phosphorylation deficiency 16; CUL3 is associated with Pseudohypoaldosteronism, type IIE). None of these genes or the rest of the genes at the interval are known to be associated with short stature. *EPHA4* is not currently a known disease causing gene. Our patient and other similar deletion patients, as well as the mouse model provide compelling evidence indicating *EPHA4* as a novel short stature gene.

**Table 2:** **The genomics and clinical presentations of previously reported deletion cases at 2q35-q36.2**

**Case ID**	**Sex**	**Age at exam (yrs)**	**Cytogenetic location of the deletion**	**stature**	**WS related features**	**Developmental issues**	**Additional features**	**Year reported or database**	**Reference**
**Case 1**	M	22	2q35-2q36.1 (221107075–222960879)	NA	NA	NA	Abnormal hands	DECIPHER 282314	https://decipher.sanger.ac.uk/patient/282314#overview
**Case 2**	M	4	2q35-q36.1 (215300000–225200000)	Smaller than 95% of his age-matched peers	WS1 (DC, CHL, HI)	MD, ID	NA	1993	[6]
**Case 3**	F	?	2q35-2q36.1 (219971907–224926273)	Short stature	WS1 (DC, HI, synophrys)	ID	NA	DECIPHER 248718	https://decipher.sanger.ac.uk/patient/248718#overview
**Case 4**	F	8	2q34-2q36.1 (213206475–222612545)	Proportionate short stature	NA	SLD, ID	Postnatal microcephaly, bifid uvula, heart abnormality	DECIPHER 281765	https://decipher.sanger.ac.uk/patient/281765#overview
**Case 5**	F	5	2q34-q36 (209000000–231,000,000)	Short stature	NA	Normal intelligence	NA	1976	[14]
**Case 6**	M	6	2q35-q36.2 (215300001–226100000)	<5 percentile	WS3 (DC, HNA, HI, SD)	Mild MD, DD, ID	Normal hearing, speech and bone age	1992	[15]
**Case 7**	M	4	2q35-q36.2 (215300001–226100000)	NA	WS3 (DC, HNA, HI, HLM, synophrys) blepharophimosis, a bulbous nose, a cupid’s bow upper lip with a short philtrum and high nasal bridge	NA	NA	1998	[16]
**Case 8**	F	4	2q35-q36.2 (215300001–226100000)	NA	WS3 (DC, HNA, HLM, Synophrys) a cupid’s bow upper lip, a bulbous nose and high nasal bridge	SLD	Myelomeningocele; small hands; rthrogryposis and camptodactyly partial subluxation	1998	[16]
**Case 9**	M	11	2q34-q36.2 (209000000–226100000)	Severe growth retardation	WS1 (DC, BNR, CHL, HNA, WF)	ID	NA	1994	[4]
**Case 10**	F	5	2q35-q36 (215300001–231000000)	Short stature	WS3 (CHL, HH, HI, BNR, DC, HNA, synophrys)	Severe DD	NA	1993	[5]
**Case 11**	F	4	2q36 (221,500,001-231,000,000)	NA	WS1 (CHL, DC, HH)	-	Medial eyebrow flare	2013	[9]
**Case 12**	F	infant	De novo 2q36 (221,500,001-231,000,000)	IUGR	Hypertelorism, hypoplastic nasal bridge with prominent nasal tip and anteverted nares.	DD	NA	1993	[17]

Not available (NA); Waardenburg syndrome (WS); dystopia canthorum (DC); congenital hearing loss (CHL), hypoplastic nasal alae (HNA); heterochromia idiris (HI); skin depigmentation (SD); developmental delay (DD); motor delay (MD); intellectual disability (ID); broad nasal root (BNR); white forelock (WF); intrauterine growth retardation (IUGR). HLM, HH.

*EPHA4* is a member of the EPH family of receptor tyrosine kinases. It was demonstrated that EPHA4 interacts directly with growth hormone receptor (GHR) and JAK2 [11]. They form a ternary complex in human cells [11]. It is conceivable that *EPHA4* haploinsufficiency causes short stature by impairing the growth hormone pathway that regulates down-stream effectors such as STAT5B and IGF1. Segregation of the short stature phenotype with *EPHA4* mutations will further support the causal relationship.

It is interesting to note that growth hormone injection did not improve the growth of the *Epha4* homozygous knockout mouse. In our patient, growth hormone replacement therapy resulted in significant improvement of growth velocity and height. It is not known if heterozygous knockout mice will respond to growth hormone or not. The response we observed in our patient provided preliminary indication for rhGH treatment in patients with *EPHA4* deletion.

## Conclusions

The rare combination of Waardenburg syndrome phenotype and short stature observed in our patient can be explained by the haploinsufficiency of both *PAX3* and *EPHA4* genes involved within the deletion. Our analysis indicated that *EPHA4* is a novel short stature gene. Recombinant human growth hormone treatment improved the height of the patient; suggesting that diagnosis may help in determining utility of growth hormone in other individuals with *EPHA4* associated short stature.

## Consent

We obtained a written consent from patient’s parent for utilizing patient‘s clinical information, genetic details and photos in this publication. The study is approved by the internal review board of the Guangxi Maternal and Child Health Hospital.

## Endnotes

^a^W index is defined as X + Y + a/b. a = inner canthal distance; b = interpupillary distance; c = outer canthal distance. A W index result greater than 1.95 is abnormal.

## Abbreviations

PAX3: Paired box 3
WS: Waardenburg syndrome
CMA: Chromosomal microarray
EPHA4: Ephrin Receptor A type 4
SD: Standard deviation
TSH: Thyroid stimulating hormone
FT3: Free triiodothyronine
FT4: Free thyroxine
FSH: Follicle stimulating hormone
LH: Luteinizing hormone
IGF-1: Insulin-like growth factor-1
MRI: Magnetic resonance imaging
rhGH: Recombinant human growth hormone
EPH: Ephrin
GHR: Growth hormone receptor
JAK2: Janus kinase 2
